# Examining Brain Activity Responses during Rat Ultrasonic Vocalization Playback: Insights from a Novel fMRI Translational Paradigm

**DOI:** 10.1523/ENEURO.0179-23.2024

**Published:** 2024-10-01

**Authors:** Lauren E. Granata, Arnold Chang, Habiba Shaheed, Anjali Shinde, Praveen Kulkarni, Ajay Satpute, Heather C. Brenhouse, Jennifer A. Honeycutt

**Affiliations:** ^1^Developmental Neuropsychobiology Laboratory, Department of Psychology, Northeastern University, Boston, Massachusetts 02115; ^2^Center for Translational Neuroimaging, Department of Psychology, Northeastern University, Boston, Massachusetts 02115; ^3^Affective and Brain Sciences Lab, Department of Psychology, Northeastern University, Boston, Massachusetts 02115; ^4^Research in Affective and Translational Neuroscience Lab, Department of Psychology and Program in Neuroscience, Bowdoin College, Brunswick, Maine 04011

**Keywords:** basolateral amygdala, bed nucleus of the stria terminalis, negative valence systems, task-based fMRI, translational neuroscience, ultrasonic vocalization

## Abstract

Despite decades of preclinical investigation, there remains limited understanding of the etiology and biological underpinnings of anxiety disorders. Sensitivity to potential threat is characteristic of anxiety-like behavior in humans and rodents, but traditional rodent behavioral tasks aimed to assess threat responsiveness lack translational value, especially with regard to emotionally valenced stimuli. Therefore, development of novel preclinical approaches to serve as analogues to patient assessments is needed. In humans, the fearful face task is widely used to test responsiveness to socially communicated threat signals. In rats, ultrasonic vocalizations (USVs) are analogous social cues associated with positive or negative affective states that can elicit behavioral changes in the receiver. It is therefore likely that when rats hear aversive alarm call USVs (22 kHz), they evoke translatable changes in brain activity comparable with the fearful face task. We used functional magnetic resonance imaging in male and female rats to assess changes in BOLD activity induced by exposure to aversive 22 kHz alarm calls emitted in response to threatening stimuli, prosocial (55 kHz) USVs emitted in response to appetitive stimuli, or a computer-generated 22 kHz tone. Results show patterns of regional activation that are specific to each USV stimulus. Notably, limbic regions clinically relevant to psychiatric disorders (e.g., amygdala, bed nucleus of the stria terminalis) are preferentially activated by either aversive 22 kHz or appetitive 55 kHz USVs. These results support the use of USV playback as a promising translational tool to investigate affective processing under conditions of distal threat in preclinical rat models.

## Significance Statement

Anxiety in humans often manifests as maladaptive responding to socially communicated threats. However, translational tools to study responses to negatively valenced social stimuli in rodents are lacking. The fearful face task or similar paradigms are used in humans to indicate ambiguous distal or indirect threat and provide valuable information about critical nodes of brain activity via functional magnetic resonance imaging (fMRI). Rats use well-characterized ultrasonic vocalizations (USVs) as indices of affective states with communicative value; therefore, we tested whether playback of affectively valenced USVs could be leveraged as socially communicated ambiguous threat during fMRI in awake rats. Results support the use of USV playback as a promising translational tool to investigate affective processing in response to social cues.

## Introduction

Animal models for the study of anxiety-related phenotypes are limited by a lack of biological markers that are reliably translatable to humans. One key domain of anxiety identified in the NIH Research Domains Criteria (RDoC) is responsivity to potential threat, described as the activity of a brain system in response to harm that may potentially occur but is distant, ambiguous, or low/uncertain in probability. From this perspective, studies of anxiety will benefit from stimuli that reliably engage neural circuits regulating the assessment of ambiguous or uncertain threats in both rodents and humans. Furthermore, translational methodologies will help to ensure biomarkers that advance to human trials have the highest possible chance of success.

Traditional paradigms in animals to assess potential threat responsivity within negative valence systems have limitations, due to the lack of translatability or relevance to human experience (Honeycutt et al., 2022). Importantly, anxiety in humans often involves maladaptive sensitivity to socially communicated threats as measured in paradigms such as fearful face presentation (Dannlowski et al., 2012; Sandre et al., 2018). Indeed, fearful and threatening facial expressions are more effective at engaging a strong and consistent amygdala response than nonface stimuli depicting fearful or threatening situations (Hariri et al., 2002). Social threat cues are of particular importance since socially communicated threats are resistant to extinction (Bublatzky et al., 2014) and mammals at all stages of development perceive socially communicated threats as relevant (Boyer and Bergstrom, 2011). Therefore, socially communicated cues can be powerful and translationally useful stimuli for studying anxiety-related circuitry. However, rodent studies typically rely either on conditioned, nonsocial stimuli such as tones that predict shock or on more proximal, nonsocial unconditioned stimuli (i.e., predator odor, bright light; Lezak et al., 2017). To more directly study affective dysfunction in preclinical models of psychiatric illness, the use of cues associated with distal or unpredictable threat are needed to dissociate fear-associated outcomes with those more indicative of anxiety (Grupe and Nitschke, 2013; LeDoux and Pine, 2016). Techniques for measuring brain activity in rodents, while advantageous with regard to cell-type specificity and high resolution, often compromise the global and translational scope of analysis that is offered by fMRI methodologies used in human research. Blood oxygenation level-dependent (BOLD) fMRI has revealed a group of brain regions that exhibit state- and trait-specific responses to fearful or angry faces, with activation of several amygdala, extended amygdala, and striatal nuclei in response to socially communicated threat (Bishop et al., 2004; Ewbank et al., 2010; Wessing et al., 2019; Zhu et al., 2023). Thus, identification of socially relevant, emotionally valenced stimuli in rats for fMRI imaging presentation can provide a translatable analog for anxiety-associated brain circuit activation to distal and/or ambiguous threat cues such as fearful faces.

While rodents lack the human capability to transfer social information via facial expression, they instead produce ultrasonic vocalizations (USVs) at distinct frequency ranges conveying danger or affiliative intent to conspecifics (Knutson et al., 2002; Panksepp and Burgdorf, 2003). USVs have been well characterized in rats and mice as indices of anticipatory affective states (Knutson et al., 2002) and notably have communicative value (Wöhr and Schwarting, 2007, 2013). When rats experience or anticipate positive affective stimuli, they emit short USV chirps typically at a frequency of ∼55 kHz (Knutson et al., 1998; Brudzynski, 2009). Conversely, rats reliably emit 22 kHz USVs across a variety of aversive or threatening situations (Kaltwasser, 1990; Blanchard et al., 1991; Tornatzky and Miczek, 1994; Covington and Miczek, 2003; Wöhr et al., 2005; Kroes et al., 2007; Mallo et al., 2009; Berger et al., 2013; Drugan et al., 2013; Fendt et al., 2018) which serve as alarm calls capable of warning conspecifics of possible danger and/or aversive situations (Blanchard et al., 1991).

A recent comprehensive review of 22 kHz playback studies in rats highlights the ability of 22 kHz USV playback to engage some of the same brain regions observed in humans presented with fearful and emotional faces (Bonauto et al., 2023). Of the regions activated by 22 kHz USV playback, two regions well documented in threat assessment—the basolateral amygdala (BLA; de Gelder et al., 2014; Michely et al., 2020) and the bed nucleus of the stria terminalis (BNST; also called the extended amygdala; Davis et al., 2010; Lebow and Chen, 2016)—showed robust increases in c-Fos activity (Sadananda et al., 2008; Ouda et al., 2016; Demaestri et al., 2019; Shukla and Chattarji, 2022; for review, see Bonauto et al., 2023). Both the amygdala and BNST are involved in processing and responding to potential threat cues, with BNST activity thought to sustain anxiety-like states (Davis, 1998, 2006). These findings overlap with human fMRI findings indicating that the presentation of fearful and/or emotional faces induce increased BOLD activity across the amygdala (Killgore and Yurgelun-Todd, 2001; Vuilleumier et al., 2001; Bishop et al., 2007) and the BNST (Sladky et al., 2018; Naaz et al., 2019). While 22 kHz playback studies in rats show changes in both the BLA and BNST, in addition to inducing anxiety-like behavior (Inagaki and Ushida, 2017; Fendt et al., 2018; Demaestri et al., 2019), almost all studies used only male subjects. Further, a more translational approach is needed to understand the nuance of USV-evoked brain activity more acutely, instead of the longer temporal timescale needed to assess c-Fos reactivity. The question also remained whether USVs could be used as stimuli to provoke hemodynamic brain responses in circuits analogous to those engaged in humans in response to socially communicated potential threat. To address these gaps in knowledge, we conducted the first proof-of-concept experiment aimed at determining the ability of affectively valenced USV playback to elicit hemodynamic responses in the BLA and BNST in awake male and female rats.

Brain activity in response to visual or auditory stimuli can only be effectively measured in awake animals, as anesthesia affects sensory, perceptive, and cognitive systems (Ferris, 2022). Therefore, we utilized an awake animal, boxcar design fMRI procedure with a well-characterized acclimation protocol (Reed et al., 2013; Ferris, 2022) and specialized fMRI-compatible ultrasonic headphones to assess whether BLA and BNST BOLD response to playback of negatively valenced 22 kHz USVs can be used as a tool to measure response to ambiguous social threat cues in comparison with appetitive social cues (55 kHz) or frequency range (22 kHz tone) controls. Here, we show that 22 kHz USV playback in awake rats evokes increased BOLD response in the BNST, comparable with findings in humans during fearful face presentation, that is not observed following control auditory cue presentations. Thus, this approach may serve as a promising preclinical assay in rat models of psychiatric disorders to assess changes in brain activity in regions associated with affective processing.

## Materials and Methods

### Subjects

Male (*n *= 24) and female (*n *= 26) Sprague Dawley rats arrived at our facilities between postnatal day 35 and postnatal day 40 (Charles River Laboratories). Rats were same-sex pair-housed under standard laboratory conditions in a temperature- and humidity-controlled vivarium on a 12 h light/dark cycle (lights on at 0700 h) with access to food and water *ad libitum*. Rats were left undisturbed for a minimum of 7 d prior to the experiment to acclimate to the new environment. All animal procedures were approved by and performed in accordance with Northeastern University's Institutional Animal Care and Use Committee's regulations. Power analyses based on effect sizes estimated from previous fMRI studies revealed that a group size of 7 yielded a power of 0.857 for a nonparametric *t* test; therefore, our design utilized 14 animals/stimulus group (7 males/7 females).

### MRI-compatible earbud development and experimental setup

**Figure 1.**
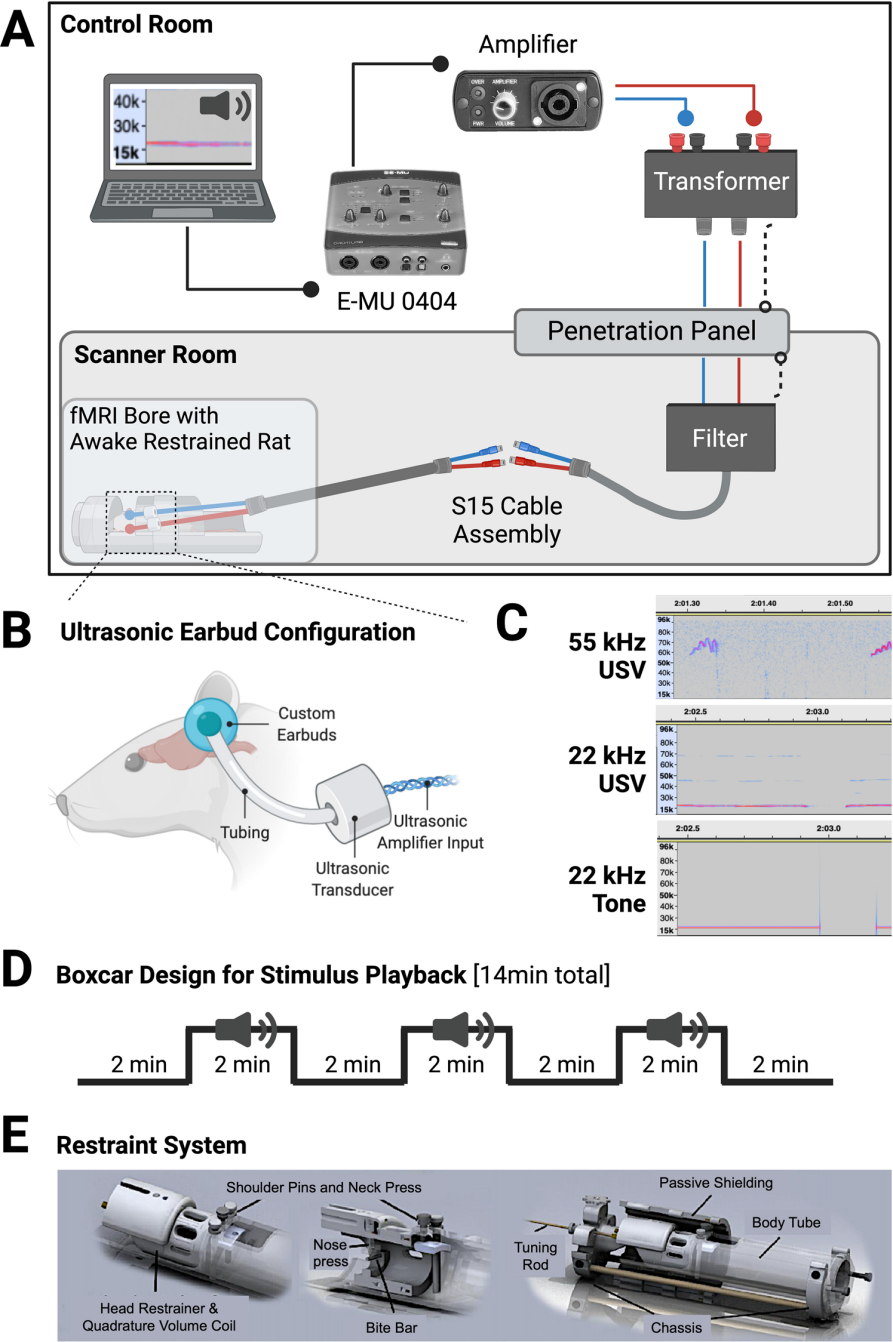
Schematic of ultrasonic vocalization fMRI playback and earbud setup. USV stimuli playback was delivered using a customized ultrasonic earbud system. The setup of the playback equipment from the control room into the scanner room and MRI bore can be seen in (***A***), with a close-up of earbud configuration (***B***). Dotted lines to the penetration panel (***A***) represent ground wires from both the transformer and filter. Spectrograms from the three playback conditions (55 kHz USV, 22 kHz USV, and 22 kHz computer-generated tone) can be seen in ***C***, and USV stimuli were delivered in a boxcar design alternating between 2 min of silence (baseline) and 2 min of stimuli (USVs or tone) for a total of 14 min (***D***) consisting of three periods of playback and 4 periods of baseline to evaluate stimuli-induced changes in BOLD signal. The 3D printed restraint system (***E***) with bite bar, head restraint, and shoulder pins, allowed for unimpeded accommodation of the earbuds and ultrasonic amplifiers while securely restraining the rat for the duration of imaging.

There are presently no commercially available MRI-compatible earbuds for rodents capable of conveying ultrasonic audio. In order to present USV stimuli to rats to determine socially valenced vocalization recruitment of target brain regions, we commissioned the production of a highly customized set of fMRI-compatible earbuds. A customized version of the 7 T compatible S15 binaural insert earphones (Sensimetrics), equipped with ultrasonic transducers and scaled down for rodent use, were created for use in the present study. Earbud configuration and additional setup considerations, including graphical depictions of the restraint system, can be seen in Figure 1.

Earbud tubing was securely fitted into the ear canals of each subject with commercially available silicone swimmer's ear putty with care taken to ensure the tubing opening remained clear of obstruction. This allowed for a customized anatomical fit for each animal, with the added benefit of dampening external noise from the fMRI in order to better deliver the auditory stimuli. Earbuds were further secured with medical tape and were reinforced when placed into the bite bar head restraint component of a quadrature transmit/receive volume coil. Tubing for the earbuds measured ∼10 cm in length between the ears and transducers, with transducers resting beside the body within the restraint tube. The ultrasonic transducers attached to cable connections that led out of the bore and connected to an S15 cable assembly and filter which was fed through a penetration panel and into a grounded S15 transformer (Sensimetrics). The transformer was connected to an ultrasonic power amplifier (#70101; Avisoft Bioacoustics) which was fed USV stimuli in .wav format from a computer through a 0404 D/A Converter Audio/MIDI interface (E-MU). Output sound fidelity was periodically verified using an UltraSoundGate Condenser microphone held up to the termination point of the earbuds with sound amplitude verified with Avisoft-SASLab Pro to ensure playback was successful and that output was averaging ∼70 dB across conditions.

### USV recordings for playback stimuli

Natural 22 kHz USVs were recorded from a restrained adult male rat while being presented with odor from cat urine. The original natural continuously recorded 22 kHz file is ∼5 min in its entirety (Demaestri et al., 2019), with 2 min of the file utilized for the boxcar design playback during functional imaging. Stimulus presentation altered between 2 min “off” (silent baseline) and 2 min “on” (USV stimulus playback) for a total of 14 min to compare change in BOLD signal from off to on states. The computer-generated 22 kHz square waveform tones were created in Audacity using the built-in tone generator tool. The number, duration, and amplitude of the tones was approximately time-matched to those in the natural 22 kHz recording. The audio for 55 kHz USVs has been used in previously published work (Wöhr and Schwarting, 2007; Demaestri et al., 2019). This audio file is ∼3.5 s in duration and consists of 55 kHz vocalizations and includes different USV categories (e.g., complex, short trills, etc.). The file was originally recorded during cage exploration with the scent of a cage mate. The 2 min audio files used in the “on” playback portion in the present study for each stimulus in the boxcar design are provided (Extended Data Audio Files 1–3). The 2 min boxcar design stimulus presentation setup was based on a prior task-based awake imaging study, where alternating 2 min conditioned stimulus presentation elicited significant changes in BOLD compared with baseline within the amygdala of experimental rats (Brydges et al., 2013). Representative spectrograms of stimuli can be seen in Figure 1*C*, along with a schematic of the boxcar design for stimulus playback (Fig. 1*D*).

10.1523/ENEURO.0179-23.2024.a1 Audio File 1 55kHz appetitive USV stimulus (attached mp4 file). Download Audio 1, MP4 file.

10.1523/ENEURO.0179-23.2024.a2 Audio File 2 22kHz aversive USV stimulus (attached mp4 file). Download Audio 2, MP4 file.

10.1523/ENEURO.0179-23.2024.a3 Audio File 3 22kHz computer-generated tone stimulus (attached mp4 file). Download Audio 3, MP4 file.

### Functional imaging system and acquisition

Imaging was conducted using a Bruker BioSpec 7.0 T/20 cm USR horizontal magnet (Bruker) with a 20 G/cm magnetic field gradient insert (ID = 12 cm) capable of 120 µs rise time. Rats were habituated to the head holder and restraining system [Ekam Imaging; see Fig.1*E* for graphical representation of restraint system; see also Ferris (2022) and Ferris et al. (2011) for additional depictions] for 5 d prior to their day of testing. During habituation days, rats were placed in the restrainer for 30 min, the maximum amount of time they would be in the scanner on the day of testing. Because the scanner generates loud noises throughout the entirety of the scan, audio recording from the scanner was played to rats during habituation so they could acclimate to the noise and environment (King et al., 2005). This method of habituation has been previously shown to be effective in decreasing stress response and possible artifacts as assessed via reduction of autonomic arousal indices (e.g., corticosterone, heart and respiration rate; King et al., 2005; Stenroos et al., 2018; Sadaka et al., 2021; Ferris, 2022).

On the day of testing, subjects were lightly anesthetized with isoflurane while being situated securely with the ultrasonic earbuds in the restraint coil system. Subjects were fully awake before scanning began. During functional scanning, each subject was presented with the primary stimulus of interest, natural 22 kHz USVs (male *n *= 8; female *n *= 7), a vocalization frequency control computer-generated 22 kHz tone (male *n *= 7; female *n *= 7), or a natural 55 kHz USVs (male *n *= 7; female *n *= 7) to serve as a social USV control. Functional MRI was performed during a 14 min session consisting of 72 min boxcar design blocks alternating between stimulus playback and silence. This resulted in three stimulus blocks and four silence blocks, which were used to compare BOLD activation within subjects. Functional MRI data was collected using a spin-echo triple-shot echo-planar imaging (EPI) sequence [imaging parameters, matrix size 96 × 96 × 20 (height × width × depth), repetition time of 1,000 ms (effective TR, 3,000 ms), echo time of 15 ms, voxel size 0.260 × 0.250 × 1.2 mm with a slice thickness of 1.2 mm, and 280 repetitions for a total acquisition time of 14 min]. These EPI scanning parameters are comparable to previously published work in rats (Honeycutt et al., 2020). Subjects’ breathing rates were continuously monitored using a Model 1025T Small Animal Monitoring & Gating System (SA Instruments) with a pneumatic pillow probe placed below the chest within the restraint with rates monitored by an experimenter throughout scanning to ensure that there were no issues with the restraint system or well-being of the animal.

### BOLD activation data analysis

Software used in the preprocessing of data files included Analysis of Functional NeuroImages (AFNI_18.3.16), Advanced Normalization Tools (ANTS_3.0.0.0), Deformable Image Registration Toolbox (DRAMMS_1.5.1), and FMRIB Software Library (FSL_6.0.3). In addition, MATLAB (MathWorks) and SPM12 (Functional Imaging Laboratory, UCL Queen Square Institute of Neurology) were used in constructing the general linear model (GLM) and second-level analysis. Functional data were first denoised due to the presence of motion spikes, which was followed by slice timing correction from an interleaved slice acquisition order. A two-step affine motion correction procedure was applied using the first volume as a reference. To improve registration, a common template was constructed to realign each subject, and affine transformations were then applied to transform each subject to this new common template. The realigned subjects were registered via two competing registration frameworks. The first was registered to the Rat Brain Atlas (Ekam Imaging) using a rigid, affine, and deformable syn (symmetric normalization) and the second via a general-purpose, deformable registration algorithm. Nuisance regression was carried out to remove signals from the white matter and cerebrospinal fluid. Finally, each subject was spatially smoothed (FWHM = 0.8 mm).

An estimation of total motion during scanning (framewise displacement) was carried out according to the method described in Power et al. (2012). Framewise displacement considers all motion due to both rotational parameters and translational; to convert rotational degrees to translational displacement, a radius of 5 mm was used. Volumes with framewise displacement >1 mm were added to a censor file to be used in the GLM. To account for motion artifacts, outlier frames were defined as those containing >3% outlier voxels, which include any voxels 3.5 times the median absolute distance after detrending with a third-degree Legendre polynomial. Any subject with 10% or more censored frames were excluded from the analysis (Table 1).

**Table 1.** Included and excluded subjects based on percent of censored frames

22 kHz USV				55 kHz USV				22 kHz tone			
Subject	#Censored frames	%Censored	Exclude?	Subject	#Censored frames	%Censored	Exclude?	Subject	#Censored frames	%Censored	Exclude?
Female_1_1	5	0.018	No	Male41_R1	0	0	No	Comp22M_R5	19	0.069	No
Female_1_2b	8	0.029	No	Female41_R2	10	0.036	No	Comp22M_R6	7	0.025	No
Female_2_1	18	0.065	No	Female41_R3	0	0	No	Comp22F_R20	5	0.018	No
Female_2_2b	48	0.175	*Yes*	Male41_R1B	7	0.025	No	Comp22M_R9	3	0.011	No
Male_3_1	5	0.018	No	Female41_R1	12	0.044	No	Comp22M_R10	5	0.018	No
Male_3_2	6	0.022	No	Male41_R3	1	0.004	No	Comp22F_R21	5	0.018	No
Male_4_1	30	0.109	*Yes*	55Male_R1	4	0.015	No	Comp22F_R22	12	0.044	No
Male_4_2	6	0.022	No	55Male_R2	51	0.185	*Yes*	Comp22M_R11	18	0.065	No
Male10b_1	10	0.036	No	55Male_R7	0	0	No	Comp22M_R12	3	0.011	No
Female10_1	0	0	No	55Female_R16	18	0.065	No	Comp22F_R23	38	0.138	*Yes*
Female11b_1	7	0.025	No	55Male_R8	14	0.051	No	Comp22M_R13	1	0.004	No
Female12_1	20	0.073	No	55Female_R17	1	0.004	No	Comp22M_R14	32	0.116	*Yes*
Female13_1	42	0.153	*Yes*	55Male_R15	7	0.025	No	Comp22F_R27	0	0	No
Male13_1	12	0.044	No	55Female_R24	6	0.022	No	Comp22F_R28	11	0.04	No
22Male_R3	3	0.011	No	55Female_R25	11	0.04	No	Comp22F_R29	11	0.04	No
22Female_R18	32	0.116	*Yes*	55Female_R30	3	0.011	No	Comp22F_R31	4	0.015	No
22Male_R4	8	0.029	No								
22Female_R19	10	0.036	No								

A GLM was constructed using a design matrix composed of the onset–offset regressor (convoluted with the hemodynamic response function), regressors due to motion-censored volumes, a constant regressor, and a linear regressor. Each voxel was then regressed using this design matrix and its betas were saved. To test our a priori hypotheses, a nonparametric one-sample *t* test was carried out for each of the three primary groups (22 kHz USV, 55 kHz USV, and 22 kHz tone). For ROI analyses, we used the “randomise threshold free cluster estimation (TCFE)” tool in the FSL software package, which was designed to specifically work with fMRI data and implements a nonparametric permutation test (Winkler et al., 2014). For a priori ROIs, BNST and BLA, we tested hypotheses using a nominal alpha < 0.05 for each test. Males and females were initially compared within each stimulus group. No sex differences were detected; therefore, males and females were pooled for all analyses.

## Results

**Figure 2.**
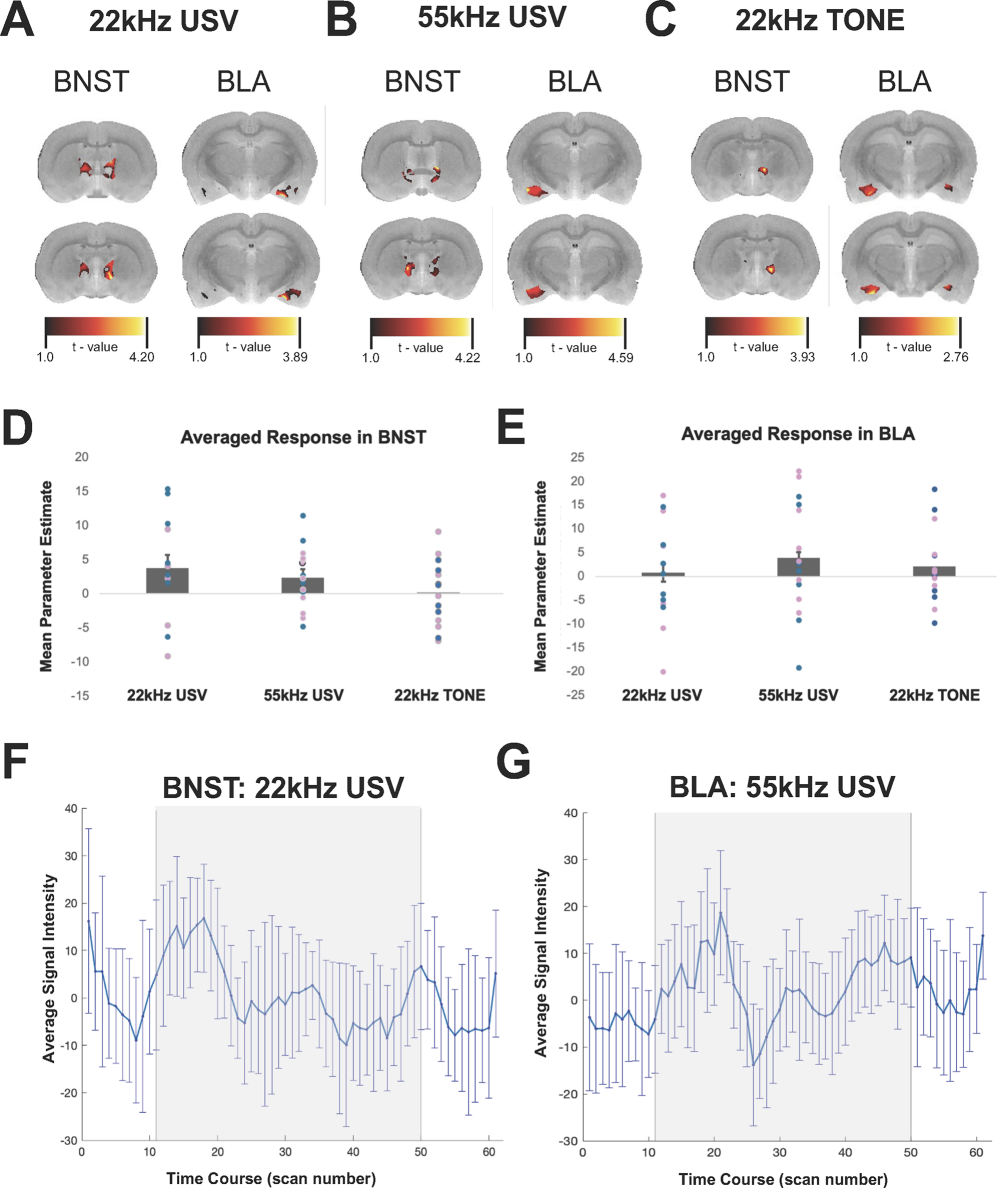
The BNST and BLA are differentially recruited in response to affectively valenced ultrasonic vocalization (USV) playback (55 or 22 kHz). Awake, restrained rats were presented with either aversive 22 kHz USVs (***A***), appetitive 55 kHz USVs (***B***), or a computer-generated 22 kHz tone (***C***) during fMRI. During aversive 22 kHz USV playback, USVs elicited significant BOLD changes from baseline in the bed nucleus of the stria terminalis (BNST). Appetitive 55 kHz USV playback significantly increased BOLD signal compared with baseline in the basolateral amygdala (BLA). Computer-generated 22 kHz tone playback elicited no significant changes in BOLD signal compared with baseline in either the BNST or BLA. Heatmaps indicate *t* statistic for BOLD signal during ultrasonic stimulus playback compared with baseline silence during scanning. Summary data for both the BNST (***D***) and BLA (***E***) are presented, with blue (male) and pink (female) dots indicating individual animal response averages within each playback condition. Time course plots of peak voxel activation for significant findings in the BNST during 22 kHz USV (***F***) and the BLA during 55 kHz USV (***G***) playback compared with silence, showing averaged signal intensity within the ROIs during playback “on” (gray shaded region) and silence “off” (flanking either side of shaded region) time points. Both summary data (***D***, ***E***) and time course plots (***F***, ***G***) are presented for descriptive purposes only.

BOLD activation by either natural 22 kHz USV, computer-generated 22 kHz tone, or natural 55 kHz USVs was determined by within-subject analysis of stimulus blocks versus silence. A cluster within the BNST showed greater activity during the 22 kHz USV audio stimulation relative to baseline (*p *= 0.041, cluster-level corrected; peak voxel *t*_(12)_ = 2.051, *p *= 0.031, one-tailed; location = [−9.36, −11, 12], *k*-extent = 9 voxels; mask volume = 1,558 voxels; Fig. 2*A*). There were no significant clusters of activity for the 55 kHz USV or 22 kHz tone stimulation conditions, relative to baseline (Fig. 2*B*,*C*).

A cluster within the BLA showed greater activity during the 55 kHz USV audio stimulation relative to baseline (*p* = 0.038, cluster-level corrected; peak voxel *t*_(14)_ = 2.084, *p* = 0.028, one-tailed; location = [−15.8, −12.4, 14.4], *k*-extent = 9 voxels; mask volume = 3,358 voxels; Fig. 2*B*). There were no significant clusters of activity for the 22 kHz USV or 22 kHz tone stimulation conditions, relative to baseline (Fig. 2*A*,*C*). The computer-generated 22 kHz tone did not significantly activate either the BLA or BNST compared with baseline. Summary figures of averaged response within the BNST (Fig. 2*D*) and BLA (Fig. 2*E*) showing individual subject points for each condition, as well as representative time course plots for significant findings—22 kHz BNST (Fig. 2*F*) and 55 kHz BLA (Fig. 2*G*)—are presented for descriptive purposes only (Kriegeskorte et al., 2009).

## Discussion

Using a novel paradigm of USV auditory playback leveraging awake rodent fMRI, we observed that brain regions associated with affective processing (BLA, BNST) were activated by differently valenced socially relevant rat USVs. Most notably, the aversive 22 kHz USV, which is emitted by rats in response to a threatening context, significantly recruited the BNST. A 22 kHz USV playback directly addresses the RDoC “potential threat” domain, which describes the pattern of responses to a distal or ambiguous threat of harm, and is typically displayed as enhanced vigilance. The evidence presented here provides a foundation for using USV playback designs—within the fMRI and more broadly—in rodent models with the ultimate goal of characterizing circuits for translational studies relevant to the treatment of psychiatric disorders in humans.

We tested the a priori hypothesis that 22 kHz USV playback would provoke BOLD activity in two nuclei that are recruited during responses to ambiguous or distal threat stimuli—the BLA and the BNST. The within-subject analysis revealed that playback of 22 kHz USVs activated the BNST, with no activation in response to a synthetic 22 kHz tone or appetitive 55 kHz USVs. These findings align with previous reports of c-Fos expression in response to natural 22 kHz USVs (Sadananda et al., 2008; Demaestri et al., 2019). Our findings further support evidence that BNST activity is preferentially recruited in response to naturalistic calls compared with artificially generated stimuli (i.e., 22 kHz computer-generated tone), despite having similar acoustic characteristics to aversive alarm calls (Ouda et al., 2016). In contrast, while the BLA did not show significant levels of activation in response to 22 kHz USVs, BOLD activity in this region was higher in response to 55 kHz USVs.

The BNST and amygdala are strongly functionally connected (Oler et al., 2012), and this connectivity is associated with trait anxiety (Brinkmann et al., 2018), making the BNST→amygdala circuit particularly important for translational studies. Specifically, both the BNST and BLA show increased activity as assessed by c-Fos expression in rodents exposed to 22 kHz USV (Demaestri et al., 2019) and as assessed with fMRI in humans exposed to fearful faces (Sladky et al., 2018; Naaz et al., 2019; note: fMRI studies report changes in the amygdala without resolution for specific nuclei). This overlapping responsivity aligns with the notion that communication between the BLA and BNST regulates behavioral response to ambiguous and sustained threats (Davis, 2006), since both aversive USV and fearful faces represent socially communicated potential danger. The BNST and BLA are also activated by exposure to predator odors (Day et al., 2004; Butler et al., 2011), supporting their role in coordinating the response to unconditioned threat stimuli. However, while the BLA projects heavily to the BNST, the BNST is particularly implicated in unconditioned responsiveness to ambiguous threats and anxiety (Hammack et al., 2015; Tovote et al., 2015; Goode and Maren, 2017) via inputs from several arousal and sensory regions. The lack of BLA BOLD response to 22 kHz USV was surprising; however, the BLA is largely implicated in circuitry important for the acquisition (Klumpers et al., 2015) and extinction (Busti et al., 2011; Giustino et al., 2020) of stimulus–response associations in relation to threat. Therefore, the lack of an associated cue or requirement for a response in the present study likely recruited less measurable BLA activity.

Presentation of prosocial 55 kHz USVs failed to activate the BNST, highlighting the specificity of our measures for stimulus valence. Activation of the BLA, however, suggests that the BLA is recruited during processing of affiliative signals and may not have been sufficiently activated during an ambiguous social threat stimulus. BLA neurons are involved in the generation of prosocial decisions (Scheggia et al., 2022), and discrete neuronal ensembles within the BLA are known to respond to either appetitive or aversive stimuli (Piantadosi et al., 2024). Since human fMRI does not have the spatial resolution to reveal BLA responsiveness to differently valenced faces, it is possible that findings from the current translational investigation highlight the capacity for the BLA to respond preferentially to positively valenced, versus negatively valenced, social stimuli in real time.

Aberrant extended amygdala activity in response to emotionally salient stimuli has been associated with anxiety disorders and major depressive disorder in youth and adults (Sheline et al., 2001; Stein et al., 2002; Killgore and Yurgelun-Todd, 2005; Kilts et al., 2006; Roberson-Nay et al., 2006; Straube et al., 2006). The magnitude of this hyperactivity is a valuable biomarker for a patient's expected response to treatment. Studies have found correlations between amygdala hyperactivation during fearful face viewing and symptom improvement after cognitive behavioral psychotherapy or medical intervention in pediatric (McClure et al., 2007), adolescent (March et al., 2004), and adult (Siegle et al., 2006) samples. On the other hand, flattened affect is a core symptom of schizophrenia (Gur et al., 2006), and patients with schizophrenia often have a reduced ability to recognize the expression of emotions in others (Kohler et al., 2000). Facial emotional recognition activates networks including the amygdala, hippocampus, visual, frontal, and thalamic regions in healthy controls, but limbic activation is diminished in patients with schizophrenia, demonstrating that emotional processing deficits may be rooted in a failure to activate these networks (Gur et al., 2007). Activation of the BNST by 22 kHz USVs is clinically relevant to psychiatric disorders with regard to aberrant socioemotional responses, supporting the utility of the USV playback paradigm in preclinical testing of pharmacological treatments in preclinical rodent models spanning anxiety, depression, and schizophrenia.

As an initial proof-of-concept experiment, this study provides preliminary evidence as to the preclinical and translational utility of this novel method of USV playback during awake fMRI to investigate affective processing. Future studies may consider modifications to the experimental design to increase robustness of results. As studies utilizing awake rodent imaging can exhibit a high degree of individual variability in signal, increasing the number of subjects per group is likely warranted. In the present study, we modeled the boxcar design in line with prior work (Brydges et al., 2013); however, the 2 min block design might have contributed to variability and modest observations. Indeed, it may be advantageous to shorten the length of each block and increase the number of on–off presentations to observe more consistent and robust signal changes. It is possible that the length of the calls in the present study, paired with the innate variability in the naturalistic 22 kHz stimulus compared with 55 kHz and tone conditions, may have masked more acute responses to the USV calls in this block design. Despite these limitations, we observed USV frequency-specific changes in BOLD activity in the BLA and BNST. These findings are in line with prior work suggesting the role of these regions in processing affective stimuli and support the ongoing optimization and utilization of this methodological approach in translational affective research.

Interpretation of the results presented here may be limited by the fact that male and female subjects were pooled in the analyses. It is well known that the expression of anxiety-like responses and their neural underpinnings may be sex dependent (Alexander et al., 2007; Michael et al., 2007; Donner and Lowry, 2013; Altemus et al., 2014; Rubinow and Schmidt, 2019; Uchida et al., 2019), but a higher-powered experiment is necessary to confirm such differences. Additionally, the rats in this study were reared in a standard laboratory environment and were not modeling any pathological condition. It is likely that more robust patterns of activation may be revealed in genetic, pharmacological, and/or behavioral models meant to recapitulate the symptomatology of psychiatric disorders, particularly those involving affective processing. Taken together, the present work builds a critical foundation for leveraging USV playback, particularly in rat preclinical models of affective dysfunction, to systematically investigate neurobiological drivers of pathology. Indeed, the present findings are the first to establish a pattern of BOLD activity in response to ambiguous social threat in typically developing rats, which may be used as a reference point for future translational studies.

## Synthesis

Reviewing Editor: Anne Keitel, University of Dundee Decisions are customarily a result of the Reviewing Editor and the peer reviewers coming together and discussing their recommendations until a consensus is reached. When revisions are invited, a fact-based synthesis statement explaining their decision and outlining what is needed to prepare a revision will be listed below. The following reviewer(s) agreed to reveal their identity: Itamar Kahn.

**Overview**: In this manuscript, the authors aimed to determine whether using the fMRI BOLD response to a socially-communicated threat (22kHz USV) had the potential to be a biomarker for preclinical studies of anxiety. Male and female rats were used to examine neural activation patterns following playback of 22 or 56kHz USVs, or a 22kHz tone. The authors report stimulus-specific (55 vs 22kHz) BOLD activity in a number of brain regions; the aversive call (22kHz USV) preferentially engaged limbic regions, while the 55kHz call (referred to as 'prosocial') resulted in higher activity in the hippocampus, medial amygdala, and somatosensory cortex. Importantly, playback of the 22kHz call led to higher BOLD activity in several anxiety-related brain regions, including the amygdala and nucleus accumbens. This work presents a novel way to examine responses to conspecific vocalizations; however, the work is highly descriptive and lacks key details that will allow assessment of scientific rigor, including under-explained experimental design choices.

**Major Concerns**

Title - The task is task-based so the title should be revised.

Abstract - Very difficult sentences to read

1. Lines 8-9 - What is meant by "translationally assessing threat responsivity to emotionally valanced stimuli" and "ambiguous potential threat"?

2. Line 20 - "psychiatric disorder neuropathology" may be redundant.

3. Line 25 - "Socially communicated threats are important negatively valanced stimuli to study mammalian anxiety ..." can be simplified and restated.

Introduction

1. Line 85 - Tone is a bit strong with the use of the words "investigators must identify"

2. Line 102 - Please clarify "playback of a 22kHz USV to male rats ..."

Methods - Overall, the methods lack sufficient details. Overall, the methods lack sufficient details.

1. Graphical depiction of the head fixation apparatus.

2. Include the assessment of head motion for all conditions and across rats.

3. Lines 156-160 - Split the text into two sentences.

4. Describe if all rats acclimated to the head state and how acclimation was verified. State whether independent measures were used to show a reduction in anxiety-like behavior. Discuss why the earbuds were not used as part of the acclimation protocol.

5. Describe how the rats' breathing rates were continuously monitored.

6. Lines 171-189 - Scan parameters were omitted. Provide an evaluation of the quality of the whole brain coverage, including temporal signal to noise ratio. Increase statistical analysis related to this.

7. Justification for the decision to present stimuli for 2 min blocks requires more detail.

8. Are the authors presenting a combination of slow (resting-state signals) and stimulus-driven responses?

9. Justification of the length of the periods is required.

10. Discuss why synthetic sounds in the prosocial and aversive groups were not provided, which could have been a more appropriate within-cohort control.

11. Discuss why rats were scanned for such short duration times.

12. Statistical analysis

a. State how many rats were excluded due to +20% censoring of volumes.

b. Specific statistical tests were not reported. State whether Wilcoxon and Mann-Whitney U as the nonparametric 1 and 2 sample t-tests were used.

c. Software package used to perform statistical analyses is unknown.

d. Report summary statistics for all different groups, both sexes, and all conditions. This will allow for evaluation of systematic differences across comparisons and whether they contribute to the reported results

e. Lack of power analyses to justify group sizes.

f. Many comparisons performed but no mention of whether the 'per comparison' p-values were adjusted to maintain the overall alpha at 0.05.

g. State whether sex differences exist using non-parametric comparisons for identified ROIs.

Results - largely minimal

1. Report the dynamics of responses (i.e., plot HRF for regions shown) and discuss if they are consistently above baseline or fluctuating.

2. Report individual rat responses, which would inform whether rats differentially show responses.

Discussion

1. Line 333 - What is referred to by "Interpretation of these ..."?

2. Line 335 - Please edit to reflect sex differences as "sexually dimorphic" and sex differences are not the same.

Legends - Overall, the legends are highly redundant with the information in the methods or results sections. Place information in 1 section and not in both.

Figures

1. Brain maps are difficult to see due to size and resolution. Increase the number of rows so that images can be larger.

2. Figure 2 - May be better remove the maps showing response to tone since few regions were activated

3. Figure 3 - Regarding labels for A and B, it may be more appropriate to use 22kHz USV > 55kHz USV as opposed to 'vs'. Also, please add a subheading above the boxes to indicate the brain region represented

**Minor Concerns**

1. Line 31 - ";" is missing

2. Line 599 - "." is missing
